# Hydrogen Separation by Natural Zeolite Composite Membranes: Single and Multicomponent Gas Transport

**DOI:** 10.3390/ma10101159

**Published:** 2017-10-06

**Authors:** Afrooz Farjoo, Steve M. Kuznicki, Mohtada Sadrzadeh

**Affiliations:** 1 Department of Chemical and Materials Engineering, 12-372 Donadeo Innovation Center for Engineering, University of Alberta, Edmonton, AB T6G 1H9, Canada; farjoo@ualberta.ca (A.F.); steve.kuznicki@ualberta.ca (S.M.K.); 2 Department of Mechanical Engineering, 10-367 Donadeo Innovation Center for Engineering, University of Alberta, Edmonton, AB T6G 1H9, Canada

**Keywords:** zeolite membrane, natural zeolite, clinoptilolite, hydrogen separation, adsorption, hydrocarbon mixture

## Abstract

Single and multicomponent gas permeation tests were used to evaluate the performance of metal-supported clinoptilolite membranes. The efficiency of hydrogen separation from lower hydrocarbons (methane, ethane, and ethylene) was studied within the temperature and pressure ranges of 25–600 °C and 110–160 kPa, respectively. The hydrogen separation factor was found to reduce noticeably in the gas mixture compared with single gas experiments at 25 °C. The difference between the single and multicomponent gas results decreased as the temperature increased to higher than 300 °C, which is when the competitive adsorption–diffusion mechanism was replaced by Knudsen diffusion or activated diffusion mechanisms. To evaluate the effect of gas adsorption, the zeolite surface isotherms of each gas in the mixture were obtained from 25 °C to 600 °C. The results indicated negligible adsorption of individual gases at temperatures higher than 300 °C. Increasing the feed pressure resulted in a higher separation efficiency for the individual gases compared with the multicomponent mixture, due to the governing effect of the adsorptive mechanism. This study provides valuable insight into the application of natural zeolites for the separation of hydrogen from a mixture of hydrocarbons.

## 1. Introduction

Employing membrane technology in gas purification offers an environmentally sustainable alternative compared with conventional thermal separation processes such as distillation [1]. Zeolites, as porous crystalline aluminosilicate minerals, have pores comparable to the kinetic diameter of the gases, which enables the separation of components by both molecular sieve properties and the difference in adsorption affinity [2,3,4]. Zeolite membranes are regenerable, and their high thermal and chemical stability make them suitable candidates for applications involving extreme conditions (e.g., chemically harsh feed streams with high temperatures) [5,6]. Compared with the synthetic zeolites, monolithic natural zeolites have experienced prolonged time and pressure that has reduced or eliminated the formation of intercrystalline grain boundaries. Hence, natural zeolites possess higher mechanical integrity than the analogue synthetic ones. However, microporous intercrystalline gaps, as a type of crystal defects, may still exist in the natural zeolites and decrease the separation efficiency of the synthesized membranes [1,4,5].

In gas separation by zeolite membranes, the separation performance primarily depends on the adsorption affinity of gas components onto the zeolite surface and their diffusion rate through the nanopores of the zeolite membrane [7]. It is well known that there is an interaction between these two phenomena, as the adsorption of gases in the channels of the zeolite influences the diffusivity of the molecules through the crystals [8]. In a multicomponent gas mixture, the competitive adsorption of gas molecules has a significant impact on the diffusion of the target permeating gas. Hence, the prediction of the separation performance of molecular sieve membranes requires knowledge of multicomponent adsorption and diffusion models in microporous materials [9]. Although extensive research has been carried out on the prediction of multicomponent gas permeation through zeolite membranes based on single gas results, the permeation of gas mixtures containing more than two components is very limited [10,11]. 

Funke et al. showed that the permeation of organic vapors through silicate zeolite membranes was strongly dependent on the presence of other species, and the pure component permeance cannot be used to predict the permeation properties of a gas mixture [12]. Using mixtures of strongly and weakly adsorbing gases, Vroon et al. showed that the gas flux through Mordenite Framework Inverted (MFI) zeolite membranes was a function of adsorption, and the diffusion of weakly adsorbing gas was suppressed by the adsorption and the mobility of the strongly adsorbing molecules due to the pore-blocking effects. Separation factors for the binary mixture of butane isomers (n-butane/isobutane) and methane/n-butane were 11 and 0.7 at 200 °C, respectively. Single-gas experimental results showed that the ideal and mixed separation factors were not pronounced for the n-butane/isobutane mixture compared with the methane/n-butane mixture [13]. For a mixture of H_2_ and CH_4_, Geus et al. obtained permeability ratios ranging from 1.3 to 15.5 through ZSM-5 (MFI-type) membranes at different feed pressures and concentrations. The permeability of hydrogen was found to increase at lower concentrations of methane, within the Henry region, so that the mixed gas H_2_/CH_4_ selectivity increased from 1.0 to 11.3 for 77/23 to 87/13 H_2_/CH_4_ gas compositions, respectively, at 21 °C and 2 atm feed pressure [14]. Hence, it was predicted that the permeability of pure H_2_ would be higher than that of a mixture of gases. This deviation from the ideal separation factor was attributed to the reduction in the diffusivity of weekly adsorbing gases (H_2_) in the presence of more adsorbing species (CH_4_). In another study, Geus et al. found that pure CH_4_ permeated 2.7 times faster than n-butane, whereas, in a 50/50 mixture, n-butane penetrated about 50 times faster than CH_4_ at 298 K [15]. Bai et al. studied the separation of H_2_/isobutane and H_2_/SF_6_ mixtures using silicalite/γ-Al_2_O_3_ membranes. The ratio of single gas permeances was 136 for H_2_ to SF_6_ and 1100 for H_2_ to isobutane at 298 K, while the largest separation factors for H_2_/isobutane and H_2_/SF_6_ were 11.9 and 12.8, respectively, which were obtained at 583 K [16].

Funke et al. showed that the permeation of organic vapors through silicate zeolite membranes was strongly dependent on the presence of other species, and the pure component permeance cannot be used to predict the permeation properties of a gas mixture [12]. Using mixtures of strongly and weakly adsorbing gases, Vroon et al. showed that the gas flux through Mordenite Framework Inverted (MFI) zeolite membranes was a function of adsorption, and the diffusion of weakly adsorbing gas was suppressed by the adsorption and the mobility of the strongly adsorbing molecules due to the pore-blocking effects. Separation factors for the binary mixture of butane isomers (n-butane/isobutane) and methane/n-butane were 11 and 0.7 at 200 °C, respectively. Single-gas experimental results showed that the ideal and mixed separation factors were not pronounced for the n-butane/isobutane mixture compared with the methane/n-butane mixture [13]. For a mixture of H_2_ and CH_4_, Geus et al. obtained permeability ratios ranging from 1.3 to 15.5 through ZSM-5 (MFI-type) membranes at different feed pressures and concentrations. The permeability of hydrogen was found to increase at lower concentrations of methane, within the Henry region, so that the mixed gas H_2_/CH_4_ selectivity increased from 1.0 to 11.3 for 77/23 to 87/13 H_2_/CH_4_ gas compositions, respectively, at 21 °C and 2 atm feed pressure [14]. Hence, it was predicted that the permeability of pure H_2_ would be higher than that of a mixture of gases. This deviation from the ideal separation factor was attributed to the reduction in the diffusivity of weekly adsorbing gases (H_2_) in the presence of more adsorbing species (CH_4_). In another study, Geus et al. found that pure CH_4_ permeated 2.7 times faster than n-butane, whereas, in a 50/50 mixture, n-butane penetrated about 50 times faster than CH_4_ at 298 K [15]. Bai et al. studied the separation of H_2_/isobutane and H_2_/SF_6_ mixtures using silicalite/γ-Al_2_O_3_ membranes. The ratio of single gas permeances was 136 for H_2_ to SF_6_ and 1100 for H_2_ to isobutane at 298 K, while the largest separation factors for H_2_/isobutane and H_2_/SF_6_ were 11.9 and 12.8, respectively, which were obtained at 583 K [16].

Given the above findings, measuring the permeation of gas mixtures with compositions and separation conditions relevant to the industrial process is essential to the practical application of the zeolite membranes. The permeation properties of a gas mixture depend on membrane material characteristics such as pore size and adsorption strength, as well as gas properties such as molecular size, shape, and composition [17,18,19]. 

In our previous study [20], we reported H_2_ separation using tubular stainless steel supported clinoptilolite membranes. The results showed high hydrogen permeance and promising H_2_/CO_2_ and H_2_/C_2_H_6_ selectivity well above Knudsen diffusion, at temperatures up to 300 °C [20]. The objective of this work is to evaluate the performance of metal supported natural clinoptilolite membranes for hydrogen separation from a gas mixture and compare it with single gas separation results. The contribution of the adsorption affinity of individual gases was investigated by conducting gas permeation and through adsorption isotherms tests at different temperatures and pressures. These results provide not only valuable insight into the practical applications of zeolite membranes in separation processes, but also an improved fundamental understanding of multicomponent gas transport in polycrystalline zeolite membranes.

## 2. Results and Discussion

### 2.1. X-ray Diffraction (XRD) Results 

**Figure 1:**
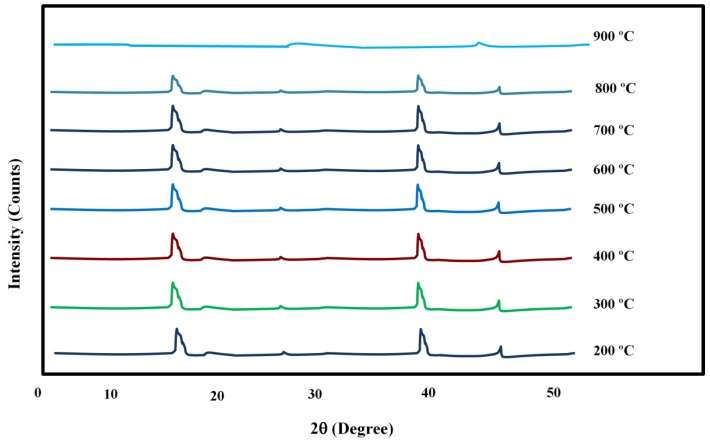
X-ray diffraction (XRD) patterns of natural clinoptilolite samples treated at 200 °C; 300 °C; 400 °C; 500 °C; 600 °C; 700 °C; 800 °C, and 900 °C for 1 h.

Figure 1 shows the x-ray diffraction (XRD) pattern of the clinoptilolite membranes heat treated at temperatures ranging from 200 °C to 900 °C. As can be observed, the natural clinoptilolite maintains a stable crystalline structure up to 700 °C. Based on the XRD results and the reported thermal stability limit by the supplier (650 °C), the permeation tests were conducted up to 600 °C to ensure that the crystalline zeolite pore structure is intact during the gas measurement tests at the studied temperature range.

### 2.2. Adsorption Parameters 

**Figure 2:**
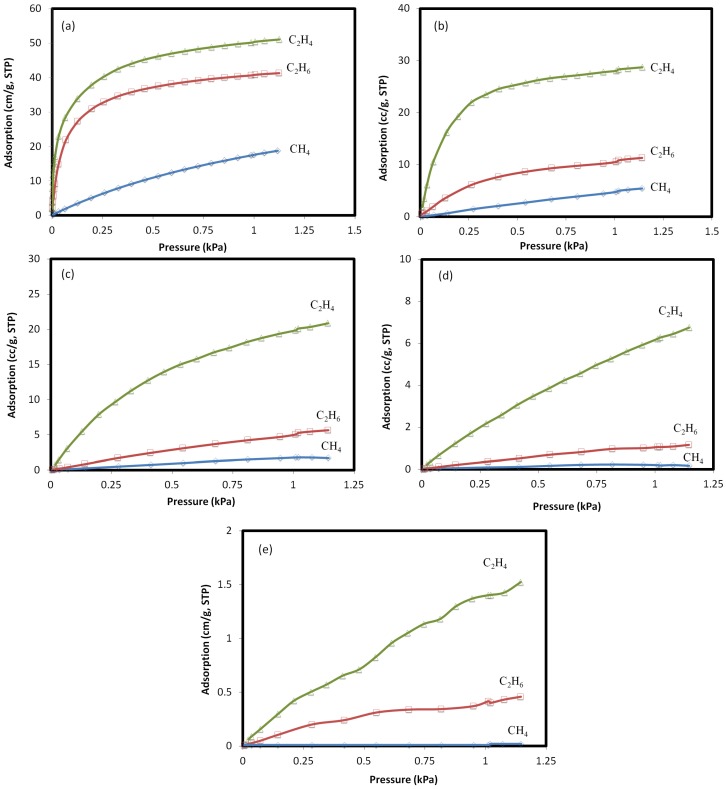
Adsorption isotherms for ethylene, ethane, and methane on natural clinoptilolite at (**a**) 25 °C; (**b**) 100 °C; (**c**) 200 °C; (**d**) 300 °C, and (**e**) 400 °C.

Adsorption isotherms for ethylene, ethane, and methane at the temperature range of 25–400 °C are shown in Figure 2. At all of the temperatures, the isotherms for natural clinoptilolite followed the capacity sequence of C_2_H_4_ > C_2_H_6_ > CH_4_. The adsorption of hydrogen on clinoptilolite could not be measured at this temperature range. The stronger adsorption of C_2_H_4_ on the zeolite is attributed to the strong quadrupole moment that results in specific interactions between the ethylene π-bonds and the cationic sites in the zeolite micropores [21,22]. The isotherms of methane and ethane are linear at temperatures higher than 25 °C, while for ethylene, the isotherm has a rectangular pattern due to the strong adsorption of this gas onto the zeolite pores. At temperatures up to 200 °C, there is a meaningful difference between the adsorption affinities of gases, which implies higher adsorptive selectivity. At higher temperatures, however, the adsorptive affinity of all of the gases decreased, and no adsorption was recorded for any of these gases at 400 °C.

According to the relative adsorption isotherms, hydrogen is the non-adsorbing or weekly adsorbing gas on clinoptilolite, ethane and methane are moderately adsorbing gases, and ethylene is a strongly adsorbing gas.

The loading inside the zeolite can be related to the partial pressure of component *i* using an adsorption isotherm model [23]. The Langmuir model was employed to calculate the adsorption parameters [24,25]. Based on this model, the fractional occupancy of the adsorption sites is calculated as follows:

(1) $$\theta_{i}=\frac{V}{V_{sat}}=\;\frac{Kp_{A}}{1+\;Kp_{A}}$$

where *V* for a given adsorbent is the amount of gas adsorbed on the solid, *V*_sat_ represents the saturation or maximum adsorption capacity, *p* is the corresponding partial pressure in the gas phase, and *K* is the adsorption equilibrium constant [26].

The adsorption parameters of the Langmuir isotherm for methane, ethane, and ethylene at 25 °C and pressures up to 115 kPa are presented in Table 1. Higher equilibrium constants in the Langmuir equation represent a higher Gibbs energy change involved in bringing a gas molecule to the surface of the adsorbent [27,28]. Hence, clinoptilolite demonstrates higher monolayer adsorption capacity for ethylene compared with ethane and methane. The high adsorption selectivity of ethylene indicates that the diffusivity of gas components is the principal mechanism for separation, rather than the molecular sieve property of the zeolite membrane.

### 2.3. Effect of Pressure 

Before conducting the permeation tests, the integrity and robustness of the synthesized membranes must be confirmed. Our earlier investigations have indicated the synthesis of defect-free mixed matrix membranes after coating with zeolite slurry [20,22]. After the formation of a zeolite layer, permeation tests were also conducted using gases of different molecular sizes, such as H_2_, CO_2_, and C_2_H_6_ to ensure the absence of large defects in the membrane. These membranes are then used in the present work to explore the effects of pressure and temperature on pure and mixed gas permeation results. 

**Figure 3:**
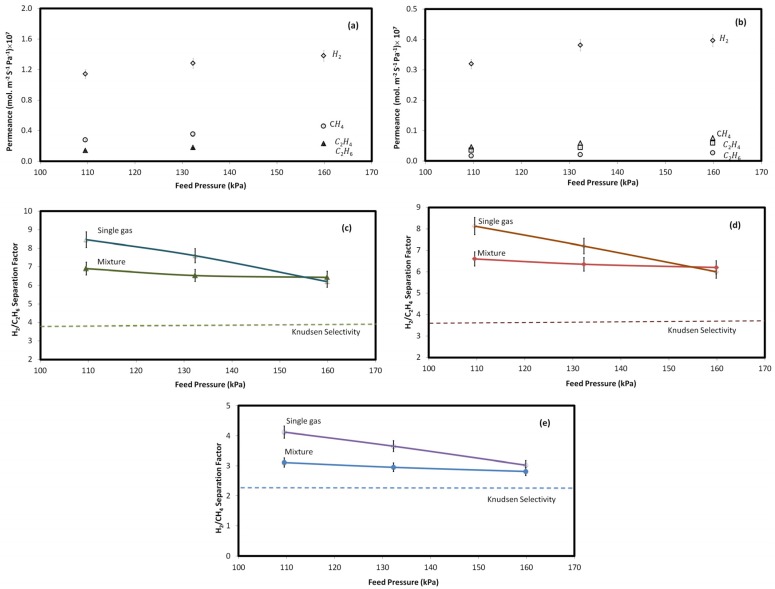
Permeance of (**a**) single gas and (**b**) mixed gas, and selectivity values for (**c**) H_2_/C_2_H_6_, (**d**) H_2_/C_2_H_4_, and (**e**) H_2_/CH_4_ in multicomponent (closed symbols) and single gas (open symbols) permeation tests at different feed pressures, as well as at constant temperature (25 °C) and permeate pressure (101.3 kPa).

To investigate the effect of pressure, single and multicomponent gas permeation tests were conducted at various feed pressures (108–165 kPa). In all of the experiments, feed temperature and permeate pressure were kept constant at 25 °C and atmospheric pressure, respectively. Figure 3a,b show the permeation results for the pure and multicomponent gases through the zeolite membrane. For both pure and multicomponent gas tests, increasing the pressure enhanced the permeance of each individual gas through the microporous membrane. Gas permeation across through zeolite membranes is typically governed by the combination of zeolitic, viscous, and Knudson transports, which are associated with both zeolite and non-zeolite pores. The decreasing trend of permeance at higher pressures can be attributed to the growing contribution from viscous flux through the relatively larger non-selective (non-zeolite) pores [14,20,29,30]. Operating pressure was found to have a larger influence on single gas permeance than multicomponent gas permeance. For instance, increasing the pressure increased pure hydrogen flux by 21%, while it only rose by about 6% in the mixed gas experiment. 

As shown in Figure 3c–e, separation efficiency decreased as the pressure increased, which indicates the presence of interstitial spaces between the zeolite crystal particles, or nonzeolitic pores contributing to Knudsen or viscous flow [20,29,30,31]. The decline in separation efficiency can also be justified by the competitive adsorption–diffusion mechanism [32,33]. Increasing pressure increases both adsorption and diffusion of gas molecules. However, the adsorption rate of highly-adsorbing gases (e.g., C_2_H_4_) is affected more significantly by pressure than the diffusion rate of non-adsorbing gases (e.g., H_2_), thereby reducing H_2_/gas selectivity. 

Figure 3 also indicates that, for both single and multicomponent gas permeation tests, H_2_/C_2_H_6_, H_2_/C_2_H_4_, and H_2_/CH_4_ separation efficiencies are higher than the corresponding Knudsen selectivities ($S_{H2,\;C2H6}^{K}={\left(\frac{{\mathrm{Mw}}_{C2H6}}{{\mathrm{Mw}}_{H2}}\right)}^{\frac{1}{2}}=3.9$, $S_{H2,\;C2H4}^{K}={\left(\frac{{\mathrm{Mw}}_{C2H4}}{{\mathrm{Mw}}_{H2}}\right)}^{\frac{1}{2}}=3.7$, and $S_{H2,\;CH4}^{K}={\left(\frac{{\mathrm{Mw}}_{\mathrm{CH}4}}{{\mathrm{Mw}}_{H2}}\right)}^{\frac{1}{2}}=2.8$), as shown by the dashed line in Figure 3. Selectivities higher than the corresponding Knudsen selectivity suggest that clinoptilolite contributed effectively to the hydrogen separation. Since Knudsen flux through microporous media remains constant as the gas pressure increases, the decrease in the trend of selectivity indicates the presence of pores that are relatively larger than the zeolite ones, resulting in viscous flow through them [17,20,34,35]. However, the actual selectivity of the membrane being higher than the Knudsen selectivity shows that the number of zeolite pores exceeds other types of pathways [36]. In fact, higher separation factors than the corresponding Knudsen selectivity suggests that the applied natural zeolite in this study contributed effectively to the separation of gases, and the intercrystalline voids were comparable to the kinetic diameter of the gases in the mixture.

Two significant differences are observed between single and multicomponent gas measurement tests. First, the average separation efficiency was higher for the single or ideal gas tests compared with the multicomponent gas experiments. Secondly, the rate at which selectivity declined with pressure was greater for the single gas than the multicomponent gas mixture. The higher separation efficiency in single gas tests is attributed to the absence of adsorptive competition exists among molecules with different sizes [37,38]. In single gas permeation tests, H_2_/C_2_H_6_, H_2_/C_2_H_4,_ and H_2_/CH_4_ selectivities declined 23.6%, 26.2%, and 26.7% with pressure (108–165 kPa), whereas, for the multicomponent gas experiment, they decreased 5.0%, 7.7%, and 6.0%, respectively. The lower impact of pressure on selectivity in the multicomponent gas blend might be attributed to the lower adsorption of highly-adsorbing gas molecules onto the zeolite surface, due to the reduced contact of these molecules with the zeolite surface.

### 2.4. Effect of Temperature 

**Figure 4:**
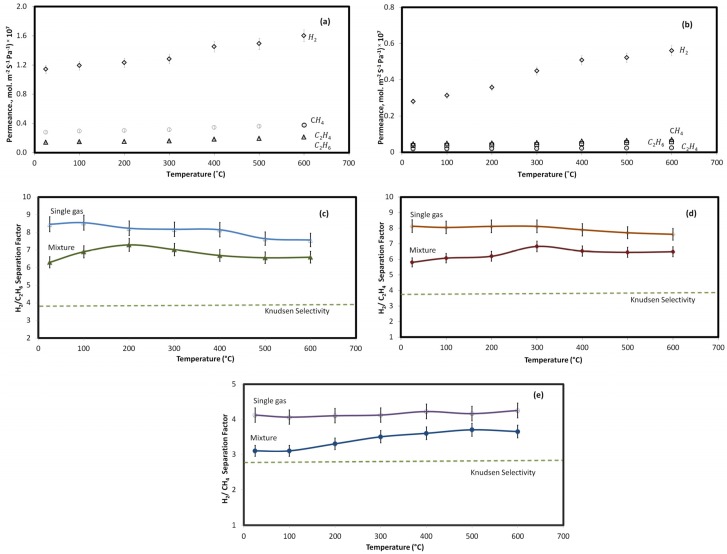
Permeance of (**a**) single gas and (**b**) mixed gas, and selectivity values for (**c**) H_2_/C_2_H_6_; (**d**) H_2_/C_2_H_4_, and (**e**) H_2_/CH_4_ in multicomponent (closed symbols) and single gas (open symbols) permeation tests at different temperatures, constant feed, and permeate pressures (111.2 kPa and 101.3 kPa, respectively).

The effect of temperature (25–600 °C) on gas permeation through the clinoptilolite membrane in both single and multicomponent gas experiments is shown in Figure 4. As shown in this figure, the zeolite membrane was stable up to 600 °C, and efficiently separates hydrogen from other gases with selectivities higher than Knudsen, $S_{H2,\;C2H6}^{K}=3.9$, $S_{H2,\;C2H4}^{K}=3.7$, $S_{H2,\;CH4}^{K}=2.8$. Figure 4a,b show the permeation results at the studied temperature range for the pure and multicomponent gases through the zeolite membrane. In single gas tests, the permeance of all gases, particularly H_2_, was enhanced upon increasing the operating temperature. As the contribution of Knudsen and viscous transports to permeance decreases with temperature, the increasing permeation trend of all of the gases as the temperature increases shows the larger contribution of zeolitic transport [14,20,22,31]. In both pure and mixed gas tests, the permeance of H_2_ was more noticeably affected than that of the other gases. This shows that, at higher temperatures, the zeolitic transport through the zeolite pores, which are comparable with the kinetic diameter of H_2_, becomes dominant. In mixed gas experiments, the adsorption of other gases, in particular C_2_H_6_, blocks the permeation of H_2_ through the zeolitic pathway at lower temperatures. 

Taking a closer look at Figure 4c–e, it is found that the separation efficiency experienced a shift in the multicomponent gas experiment at a temperature around 300 °C. As the pore size of the zeolite membrane accommodates hydrogen as the permeating molecule in the mixture, the gas separation at room temperature to 300 °C was controlled by competitive adsorption–diffusion mechanism. The adsorbing gases in the mixture were preferentially adsorbed onto the internal pore surfaces and permeated through the membrane via surface diffusion [32,33]. The shift in the permeation of the gases also occurred at 300 °C where the isotherms indicated no competitive adsorption of the gas molecules. The adsorbed gas molecules considerably reduce the free space of the membrane pores that limit the entry and passage of non-adsorbing molecules [37,39]. Hence, temperature increase causes a shift in separation mechanism that affects the separation efficiency [39,40]. At low temperature, in the presence of competitive adsorption, ethylene, as the stronger adsorbing gas, blocked zeolite pores for the permeation of hydrogen molecules, and as a result, selectivity decreased. It is worth mentioning that the competitive adsorption did not result in a selective transport of C_2_H_4_ vs. H_2_; that is, C_2_H_4_/H_2_ selectivity >1. A possible explanation is that although ethylene blocks some of the pathways of hydrogen (zeolite pores), there are still enough zeolite pores to accommodate hydrogen. It might also indicate that in the competitive adsorption–diffusion mechanism for the transport of a gas mixture through zeolite membranes, the diffusion of smaller gas molecules is dominant. An increase in the temperature made either Knudsen diffusion or activated diffusion mechanism dominant [41,42].

### 2.5. Average Defect Size

**Figure 5:**
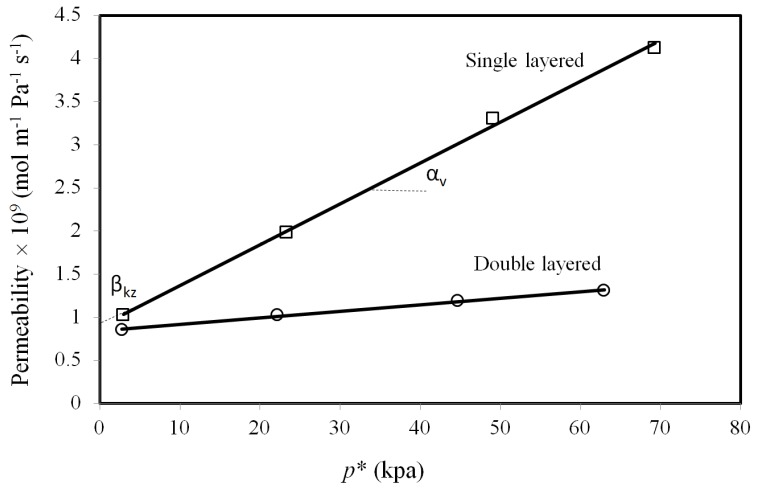
Comparative parameters (*α*_v_, *β*_kz_) for single and double-layered membranes. Permeate pressure: 108.2 kPa and temperature: 25 °C.

Figure 5 shows the permeability of single-layered and double-layered membranes as a function of p*, as defined in Equation (7). The slope and intercept of the linear fitting of this data are a pressure-dependent term (*α*_v_), and a pressure-invariable term (*β*_kz_), which are representative of “Poiseuille or viscous” non-zeolitic and “Knudsen and zeolitic” contributions, respectively. Based on the literature, of *λ* = *α*_v_/*β*_kz_ ratio can be used as a criterion to compare the defect size in zeolite-based membranes [29,43]. Membranes with the smaller non-zeolite pore size have the lower *α*_v_/*β*_kz_ ratio. According to Figure 5, the double-layered membrane shows a higher permeability, as the intersection with *y*-axis is larger. The corresponding values of *λ* = *α*_v_/*β*_kz_ for each membrane are listed in Table 2. Two major findings that can be concluded from these values are as follows: first, the *α*_v_/*β*_kz_ ratios in this study are in the range of literature values for typical zeolite membranes. Second, the single-layered membrane shows a higher value of *λ* ratio than the double-layered membrane, which means that the average defect size is slightly larger for the single-layered membrane than for the double-layered one. A larger average defect size represents higher non-zeolite fluxes for CO_2_ and C_2_H_6_ as feed pressure increases. The extent of the “non-selective” viscous flux passing through the relatively larger non-zeolite pores increased as the total pressure difference rises. This is consistent with a larger defect size for single-layered tube as compared with double-layered one:

## 3. Materials and Methods 

### 3.1. Materials 

The membrane material used in this study was a sample of clinoptilolite, Ash-Meadows, with 99% purity provided by St. Cloud Mining Company (Winston, NM, USA) with particle size corresponding to the 325 mesh for clinoptilolite, as reported elsewhere [20,30]. Porous 316L stainless steel tubes supplied by Graver Technologies (Newark, DE, USA) were used as porous substrates. The tubes were coated with a TiO_2_ layer, which was subsequently sintered to reduce the average pore size of the tube to 0.02 µm. 

The slurry that used for the coating of the stainless steel tubes was a mixture of clinoptilolite powder and an aluminosilicate solution (ALS, Accumet Materials, Ossining, NY, USA) as a binder. The binder is used to provide effective cohesion among zeolite particles and adhesion between the slurry and the metallic substrate. ALS is a standard binder for ceramic coatings. Its similar chemical properties to zeolites endow a strong chemical bonding between the binder and zeolite.

Pure and multicomponent gas permeation experiments were conducted using H_2_, C_2_H_6_, C_2_H_4_, and CH_4_ gases supplied by Praxair Canada. A gas mixture of H_2_ (35%), CH_4_ (6%), C_2_H_4_ (33%), and C_2_H_6_ (26%) was provided by NOVA Chemicals Co. in Calgary, AB, Canada.

### 3.2. Membrane Preparation Method 

Before coating the zeolite and binder slurry, stainless steel tubes were washed at room temperature using an alkaline detergent solution (Decon Labs, USA), and rinsed with distilled water in an ultrasonic bath for 1 h to remove any surface residues.

Zeolite-coated stainless steel tubular membranes were prepared using a slurry of 25 wt % clinoptilolite powder, 50 wt % Aluminosilicate binder, and 25 wt % distilled water. The slurry was homogenized by a planetary ball mill machine (Laval Lab, Canada) at 300 rpm for 20 min. A syringe pump was used to inject the coating slurry into the bottom of a vertically positioned porous stainless steel tube at a rate of 7 mL/min. The syringe was attached to the tube using a flexible rubber tube. When the tube was filled with slurry, the syringe pump was stopped, and the excess slurry was drained out by removing the rubber tube from the bottom of the porous tube. Coated tubes were air-dried at room temperature and atmospheric pressure, and finally thermally sintered in a muffle furnace at 371 °C at 2 °C/min for 4 h. More details on preparation and the membrane microstructure are provided elsewhere [20].

**Figure 6:**
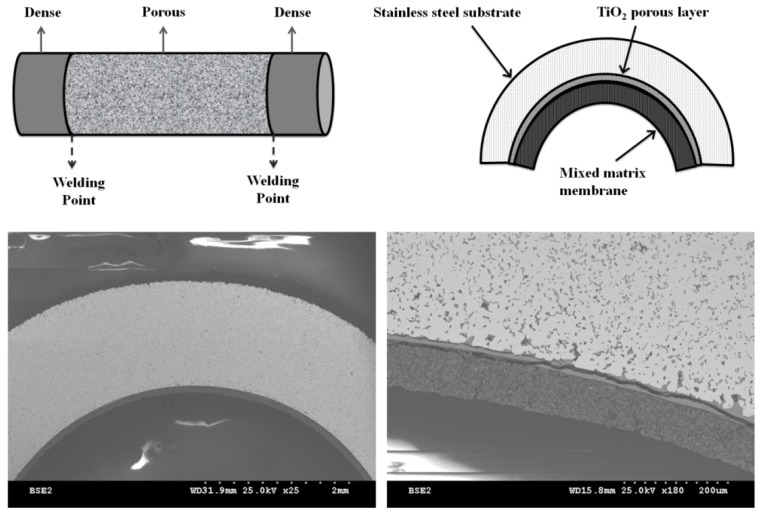
Schematic picture of side and cross-section of the tubular zeolite membrane and SEM cross-sectional images.

Following the same procedure, coated tubes were subjected to a second coating. The thickness of the resulting single and double-layered membranes was 50 and 80 µm, respectively. More detailed information about the preparation process and the corresponding characterization methods are provided in our previous study [20]. The XRD and SEM results confirmed that the blending and sintering of the zeolite layers on the surface of the porous substrate did not change the zeolite membrane crystalline microstructure. Permeance data using different molecular size gases also showed superior separation performance compared with Knudsen selectivity. This result confirmed that the number of zeolite micropores outnumbered the non-zeolite ones [20]. The schematic of the coated stainless steel (316L) tubular composite membrane (side and cross-section views) along with their SEM cross-sectional images are shown in Figure 6.

### 3.3. Characterization Methods

#### 3.3.1. X-Ray Diffraction (XRD)

To investigate the thermal and structural stability of the membrane material at different temperatures, powder samples were analyzed by XRD. The XRD patterns were collected by a Rigaku Geigerflex Model 2173 diffractometer with a cobalt Co Kα radiation source (*λ* = 1.79021 Å) ran at 2*θ* range of 5° to 90°.

#### 3.3.2. Adsorption Isotherms

Single gas adsorption experiments were carried out to determine the adsorption affinity of each component in the gas mixture on the natural zeolite. Ethane, ethylene, and methane adsorption isotherms were measured in the temperature and pressure ranges of 25–400 °C and 0–120 kPa, respectively, using Micromeritics Accelerated Surface Area and Porosimetry system (ASAP 2020C, Norcross, GA, USA) at chemisorption configuration. 

Samples of natural clinoptilolite zeolite were activated using high-temperature nitrogen flow (200 mL/min flow rate and 350 °C temperature) for 15 min. The samples were first evacuated (10–4.0 Pa) for 60 min before cooling down to 25 °C under vacuum, then dosed with fixed quantities of each gas (methane, ethane, and ethylene) until a pressure of 114 kPa was reached. The isotherm data at different temperatures (25–400 °C) were obtained by measuring the adsorptive capacity of the zeolite.

#### 3.3.3. Gas Permeation Tests 

**Figure 7:**
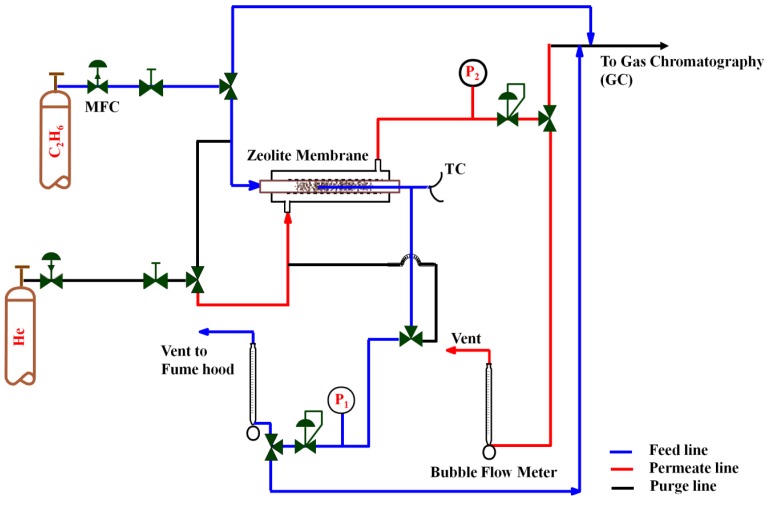
Schematic of the set-up for the gas permeation measurement.

Both single and multicomponent gas permeation tests were conducted using the set-up shown in Figure 7. The membrane was sealed in a stainless steel shell-and-tube chamber with feed and permeate (along with sweep gas) gases passed through the tube and shell sides, respectively. For permeation tests at higher temperatures, the membrane cell was placed into a tubular furnace with a multipoint programmable temperature controller. A heating rate of 5 °C/min was used to reach a specified temperature. Both single gas and multicomponent permeation tests were carried out in a feed pressure range of 110–160 kPa and a temperature range of 25–600 °C. 

The transmembrane pressure was controlled using a backpressure regulator located at the feed side outlet. The feed and sweep gas flow rates were controlled by two mass flow controllers (Sierra Instrument Inc., Monterey, CA, USA). For all of the gas permeation tests, the flow rates of feed and sweep gas were constant at 100 mL/min (STP). The flow rate of outlet streams was measured using bubble flowmeters. A Shimadzu Gas Chromatograph GC-14B (Kyoto, Japan) equipped with a thermal conductivity detector (TCD) and packed column (HaySep Q, 80–100 mesh) was used to analyze the permeate and retentate concentrations. 

The TCD senses the variation in the thermal conductivity of a gas stream when heated and compares it with a reference carrier gas, most commonly He and Ar. Hence, the difference between the thermal conductivity of the analyte and the carrier gas determines the sensitivity of the instrument. The thermal conductivity of different gases is presented in Table 3. According to this table, to achieve the maximum thermal conductivity difference between the gas chromatography (GC) carrier gas and the analyte in the single gas permeation tests, helium was used as the GC carrier gas for C_2_H_4_, C_2_H_6,_ and CH_4,_ while argon was used for H_2_ analysis. For the multicomponent gas permeation tests, however, the GC was recalibrated to detect all of the gases at the same time using argon as the GC carrier gas.

In order to evaluate the separation performance of membranes in single and multicomponent gas separation tests, the membrane selectivity must be measured. For single gas permeations, the ideal selectivity of species *i* over *j* (*S_ij_*) was calculated using the following equation:

(2) $$S_{ij}=\frac{P_{i}}{P_{j}}$$

where $P_{i}$ and $P_{j}$ are the permeance (mol·m^−2^·s^−1^·Pa^−1^) of components *i* and *j*, respectively. The gas permeance is calculated as follows:

(3) $$P_{i}=\frac{N_{i}}{\Delta p_{i}}$$

where *N_i_* is the molar flux (mol^−1^·m^−2^) and $\Delta p_{i}$ is the partial pressure difference (Pa) of component i across the membrane.

For multicomponent gas experiments, it is more common to describe the permeation driving force in terms of a fugacity difference rather than a partial pressure difference, due to the non-ideal gas behavior of gas mixtures. Separation factor for the gas mixture is given by the following equation [45]:

(4) αi=yAyBxAxB

where *x*_i_ and *y_i_* are mole fractions of component i on the feed and permeate sides, respectively, and are measured by GC. When one or two strongly adsorbing components are involved, there is no correlation between the ideal selectivity and the separation factor [26,46]. In this case, single gas experimental results deviate significantly from the multicomponent gas results [5,45].

#### 3.3.4. Relative Average Defect Size

Zeolite membranes could be screened based on the relative average defect size through using a comparative coefficient obtained when H_2_ single permeability is plotted as a function of pressure [29]. Gas transport through zeolite membrane is due to the combined contributions from diffusion through zeolite cavities and Knudsen and viscous flow through non-zeolite pores:

(5) $$N_{i,t}=\alpha N_{i,z}+\left(1-\alpha \right)N_{i,nz},\;\alpha =\frac{A_{z}}{A_{t}}$$

where $N_{i,t}$, $N_{i,z}$, and $N_{i,\mathrm{nz}}$ are total molar flux, flux through zeolite cavities and flux through non-zeolite pores, respectively (mol·m^−2^·s^−1^). $A_{z}$ and $A_{t}$ are the zeolite pore area and the total active surface area of membrane, respectively. Hence, 1−α is the fraction of the cross-sectional area corresponding to non-zeolite pores or defects. The permeability of a gas through the zeolite membrane is calculated as follows:

(6) $$\mathrm{Permeability}=\frac{N_{i,t}}{{\Delta p}_{i}}\delta =\alpha_{v}\left[p^{*}\right]+\beta_{kz}$$

(7) $$p^{*}=p_{m}\frac{\Delta p}{{\Delta p}_{i}}$$

where δ is the thickness of the membrane (m), and $\Delta p_{i}$ is the partial pressure difference of component *i* (Pa) between the feed and the permeate side, and $p_{m}$ is the mean pressure between the feed and the permeate side. According to Equation (6), H_2_ permeability across the membrane can be considered as a combination of two fractions. One fraction, the first term on the right hand side of Equation (6) (*α_v_*), which is associated with Poiseuille or viscous flow, is dependent on pressure, while the other fraction (*β_kz_*) is essentially not correlated with pressure variation, and includes Knudsen and zeolitic flux contributions. The full expression of coefficients *α_v_* and *β_kz_* are provided elsewhere [29]. These coefficients can be obtained by plotting the permeability as a function of p*, and finding the slope and intercept of a linear fitting this plot (Figure 5). The ratio *λ* = *α*_v_/*β*_kz_ is a comparative parameter that provides valuable information regarding the averaged defect size of porous membranes. The smaller the averaged non-zeolite pore, the lower the value of the *α*_v_/*β*_kz_ ratio. The values for the coefficient *λ* were estimated and compared after depositing the first and second layer of the zeolite in order to evaluate the effectiveness of the coating.

## 4. Conclusions

Geomorphic natural zeolite membranes have shown promise in the separation of H_2_ from light hydrocarbons. Permeation measurements through natural clinoptilolite membranes were performed at temperatures ranging from 25 °C to 600 °C and feed pressures from 110 to 185 kPa. The hydrogen/hydrocarbon selectivity based on a single component permeation ratio deviated significantly from mixed gas results. Besides molecular sieve property and the diffusion rate of gas molecules in a zeolite, the difference in the adsorption of gases onto the surface was found to be a key factor in separation performance. Comparison of the single gas and multicomponent gas behavior suggests that the permeation behavior of a component can be influenced by the presence of moderately or strongly adsorbing components in a mixture. Although competitive adsorption can be modeled for the zeolite systems to provide some insights into the permeation of a gas mixture through the membrane, flow through defects will not be subjected to the same forces, which affecting the predictability of the membrane performance. Therefore, the most reliable method to evaluate the membrane performance is to test them under conditions comparable to the real indutrial applications.

## Figures and Tables

**Table 1:** Adsorption parameters of the Langmuir isotherm at 25 °C of the gas mixture.

Component	$V_{sat}$ K cc/(g bar)	$V_{sat}$ cc/g	K
CH_4_	5.22	15.2	0.041
C_2_H_6_	21.5	6.13	3.54
C_2_H_4_	243.9	24.4	16.0

**Table 2:** Comparative parameter (*λ* = $\alpha_{v}$/$\beta_{\mathrm{kz}}$) for single-layered and double-layered tubing membranes.

Membrane	λ × 10^2^ (kPa^−1^)
Single-layered membrane	5.2
Double-layered membranes	0.9

**Table 3:** Thermal conductivity of gases at 27 °C [44].

Gas	Thermal Conductivity (mW/m·K)
N_2_	26.0
Ar	17.9
H_2_	186.9
He	156.7
C_2_H_4_	20.5
CH_4_	34.1
C_2_H_6_	21.3
